# An assessment of cross reactivity with patients allergic to birch pollen using the immunoblotting

**DOI:** 10.1186/2045-7022-1-S1-P93

**Published:** 2011-08-12

**Authors:** Magdalena Zbikowska-Gotz, Andrzej Ku¿miñski, Jolanta JóŸwiak, Katarzyna Napiórkowska, Iwona Fares, Ewa Socha, Jolanta Rêdowicz, Zbigniew Bartuzi

**Affiliations:** 1 Collegium Medicum in Bydgoszcz, Department of Allergology, Immunology and Internal Diseases, Bydgoszcz, Poland; 2 Polisch Science Academy, Institut of Biochemistry and Experimental Biology, Warsaw, Poland

## The aim

Evaluation of the applicability of immunoblotting for diagnosing cross reactivity between homologous proteins present in various sources. Method and material: Group of 21 patients with birch pollen allergy aged 41,7±5,2 revealing symptoms after eating cross-reacting food and positive skin tests, or prick by prick tests with native food allergens. Control group - 7 persons aged 39,8±4,7, allergic to birch pollen without any symptoms after having eaten the above mentioned food. Concentration of asIgE for birch pollen, apple, carrot and celery allergens has been measured with EIA method. Concentration of asIgE against recombinant allergens of birch pollen Bet v1, Bet v2 and celery Api g1 has been evaluated with FEIA-UniCAP 100.

As plant material served specially prepared extracts of apples', separately peel and pulp, as well as of carrot and celery. The detection of proteins following the immunobloting was conducted by incubating in the darkness the nitrocellulose membrane in a buffer solution and peroxidase substrate.

## Results

Within the group of 21 patients, 16 have developed (76,2%) positive Bet v1 reaction, include 4 subjects this reaction was less significant. The presence of IgE for Mal d1 in apples fruit was diagnosed in 14 patients (66,6%), in 3 it was weaker, whereas 7 persons (33,3%) have not revealed any reaction. Antibodies against Mal d3 from the apple peel extract was detected in serum of 6 patients (28,65%). The simultaneous presence of IgE against Mal d1 and Mal d3 proteins was detected in 6 patients. 11 subjects (53,4%) revealed the presence of sIgE for Api g1, in 4 patients the reaction was weak, and only 1 person (4,75%) had IgE for Dau c1. Within the control group, the presence of IgE against birch proteins has been detected in 6 patients, to apple - in 3, to celery – in 2, to carrot – in 1, which may correspond to Bet v1, Mal d1, Api g1, and Dau c1. Within this group, 1 patient was positive to protein of <10kDA of apple peel extract. Positive signal in immunobloting to proteins of 17-18 kDa present in all examined extracts was detected only in 1 person from patients group as well as 1 from control group.

